# Supplementary material for: Charting the Cannabis plant chemical space with computational metabolomics
Source: Metabolomics. 2024 May 25;20(3):62. doi: 10.1007/s11306-024-02125-y (PMC11127828; doi:10.1007/s11306-024-02125-y)
Supplement: Supplementary file 1 — Supplementary Material 1 [file 11306_2024_2125_MOESM1_ESM.pdf]

Metabolomics
Supplementary Figures, Tables and GNPS job links

Charting the Cannabis plant chemical space with computational metabolomics

Akhona Myoli[1], Mpho Choene[1], Abidemi Paul Kappo[1], Ntakadzeni Edwin Madala[2], Justin J.J. van der Hooft[1,3*], Fidele Tugizimana[1,4,5*]

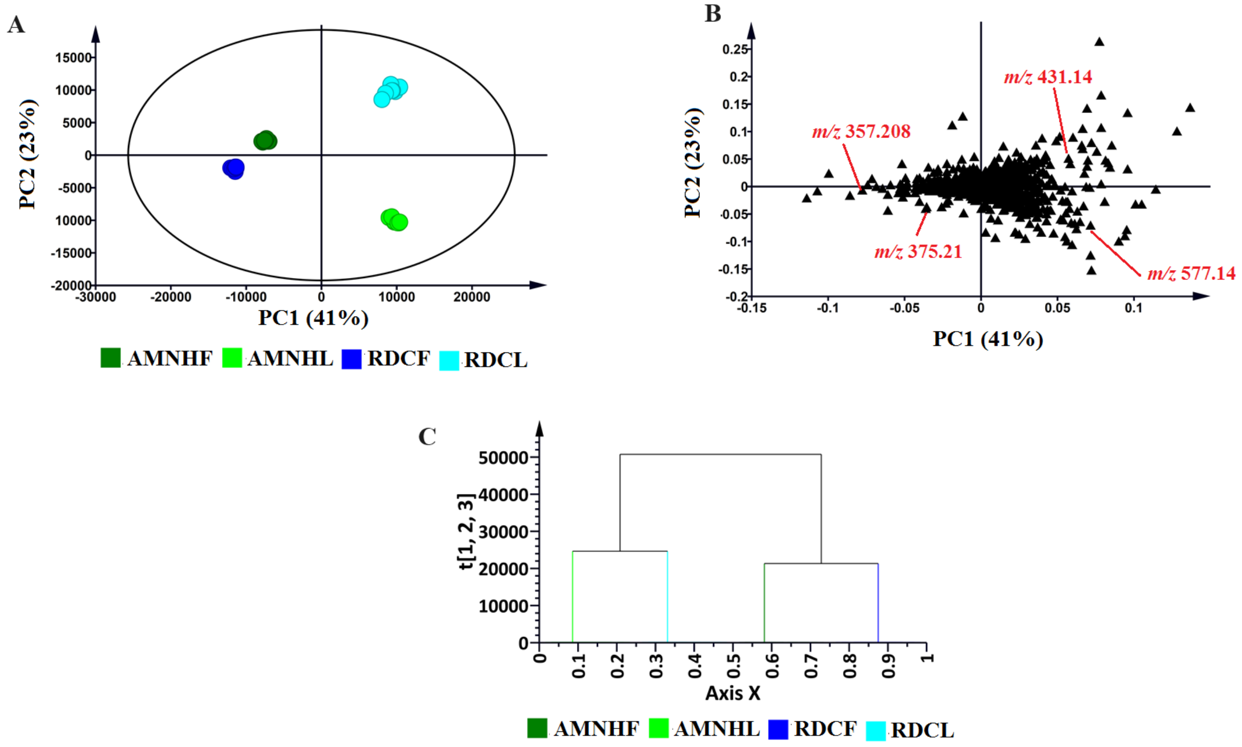

Figure S1: **Chemometrics modelling**. Unsupervised analysis and models of the ESI negative data of cannabis cultivars-Amnesia haze and Royal dutch cheese. **Key**: Amnesia haze leaves (**AMNHL**), Amnesia haze flowers (**AMNHF**), Royal dutch cheese leaves (**RDCL**) and Royal dutch cheese flowers (**RDCF**). (**A**) PCA scores plot and (**B**) loading score plot of the Pareto-scaled dataset of the two cultivars. The variables highlighted on the loadings plot are 6,7-epoxy-Cannabigerolic acid (*m/z* 375.21), cannabidiolic acid (*m/z* 357.208), vitexin (m/z 431.14) and vitexin-2-O-rhamnoside (577.14). - (**C**) The HCA dendrogram indicating the hierarchical structure of the dataset. The PCA score plot explains R2X = 78%% variation and Q2 = 74% predictive variation and the HCA corresponds to the variation shown on the PCA scores plot based on seven-fold cross-validation.

GNPS molecular networking outputs (job links) for leaves (AMNHL and RDCL) and flowers (AMNHF and RDCF):

FBMN of leaves ESI (-): ID=8cd7fb7a5f5c40e3881566fd2f11bc7d
https://gnps.ucsd.edu/ProteoSAFe/status.jsp?task=8cd7fb7a5f5c40e3881566fd2f11bc7d

FBMN of leaves ESI (+): ID=0e4fabaf54af45a9bc436f209efe5290
https://gnps.ucsd.edu/ProteoSAFe/status.jsp?task=0e4fabaf54af45a9bc436f209efe5290

FBMN of flowers ESI (-): ID=0cd602dc592f460c97a5111d2000a5eb
https://gnps.ucsd.edu/ProteoSAFe/status.jsp?task=0cd602dc592f460c97a5111d2000a5eb

FBMN of flowers ESI (+): ID=6b7f1c180143486692d975e285dcbd58
https://gnps.ucsd.edu/ProteoSAFe/status.jsp?task=6b7f1c180143486692d975e285dcbd58

Combined FBMN ESI (-): ID=a268bab486d5414cb0e716f66fd37781
https://gnps.ucsd.edu/ProteoSAFe/status.jsp?task=a268bab486d5414cb0e716f66fd37781

FBMN of leaves + vitexin standard ESI (-): ID=607fce1557fb46afbeadc69883f5f0df
https://gnps.ucsd.edu/ProteoSAFe/status.jsp?task=607fce1557fb46afbeadc69883f5f0df

FBMN of flowers + vitexin standard ESI (-): ID=9155f3807fd745788840e2315d1192a1
https://gnps.ucsd.edu/ProteoSAFe/status.jsp?task=9155f3807fd745788840e2315d1192a1

DEREPLICATOR + ESI (-) of leaves: ID=943eba0bdc69478b863c22afb7b365dd
https://gnps.ucsd.edu/ProteoSAFe/status.jsp?task=943eba0bdc69478b863c22afb7b365dd

DEREPLICATOR + ESI (+) of leaves: ID=3f00c9f413a9402c9bf713a2b294bd2c
https://gnps.ucsd.edu/ProteoSAFe/status.jsp?task=3f00c9f413a9402c9bf713a2b294bd2c

DEREPLICATOR + ESI (-) of flowers: ID=d6d817f90bc9494e9c5e0f87d477bd1c
https://gnps.ucsd.edu/ProteoSAFe/status.jsp?task=d6d817f90bc9494e9c5e0f87d477bd1c

DEREPLICATOR + ESI (+) of flowers: ID=9ad720b8ec3c40d5abda2615c98c3365
https://gnps.ucsd.edu/ProteoSAFe/status.jsp?task=9ad720b8ec3c40d5abda2615c98c3365

NAP ESI (-) of leaves: ID=378071d9403f44418b7594d6a91d4237
https://gnps.ucsd.edu/ProteoSAFe/status.jsp?task=378071d9403f44418b7594d6a91d4237

NAP ESI (+) of leaves: ID=28f6d06b4cf84950971645ab4fbf281f
https://gnps.ucsd.edu/ProteoSAFe/status.jsp?task=28f6d06b4cf84950971645ab4fbf281f

NAP ESI (-) of flowers: ID=4e851eb2d2524496b1a65c492eeeb784
https://gnps.ucsd.edu/ProteoSAFe/status.jsp?task=4e851eb2d2524496b1a65c492eeeb784

NAP ESI (+) of flowers: ID=7011cdd48fb74bb487ce4db82e8bcb3a
https://gnps.ucsd.edu/ProteoSAFe/status.jsp?task=7011cdd48fb74bb487ce4db82e8bcb3a

MS2LDA ESI (-) of leaves: ID=3ccc5c471c3041c1acc3fce8fc397856
https://gnps.ucsd.edu/ProteoSAFe/status.jsp?task=3ccc5c471c3041c1acc3fce8fc397856

MS2LDA ESI (+) of leaves: D=8cc3b956bcd24c15bcd00f0be4afc17c
https://gnps.ucsd.edu/ProteoSAFe/status.jsp?task=8cc3b956bcd24c15bcd00f0be4afc17c

MS2LDA ESI (-) of flowers: ID=1684184ee24f47af9b812f12addaf821
https://gnps.ucsd.edu/ProteoSAFe/status.jsp?task=1684184ee24f47af9b812f12addaf821

MS2LDA ESI (+) of flowers: ID=f71509557b4942b1ab147ed843fb63d7
https://gnps.ucsd.edu/ProteoSAFe/status.jsp?task=f71509557b4942b1ab147ed843fb63d7

MS2LDA ESI (-) of leaves + vitexin standard: ID=29027ad86fa84025aa73df572862beae
https://gnps.ucsd.edu/ProteoSAFe/status.jsp?task=29027ad86fa84025aa73df572862beae

MS2LDA ESI (-) of flowers + vitexin standard: ID=52bc3bbf53504151acfab5e167a3ef60
https://gnps.ucsd.edu/ProteoSAFe/status.jsp?task=52bc3bbf53504151acfab5e167a3ef60

MolNetEnhancer ESI (-) of leave: ID=d65b3a5b5e7949b28f3ac672b59b2c60
https://gnps.ucsd.edu/ProteoSAFe/status.jsp?task=d65b3a5b5e7949b28f3ac672b59b2c60

MolNetEnhancer ESI (-) of flowers: ID=e9634b2290f04e1bbd976a2e99866822
https://gnps.ucsd.edu/ProteoSAFe/status.jsp?task=e9634b2290f04e1bbd976a2e99866822

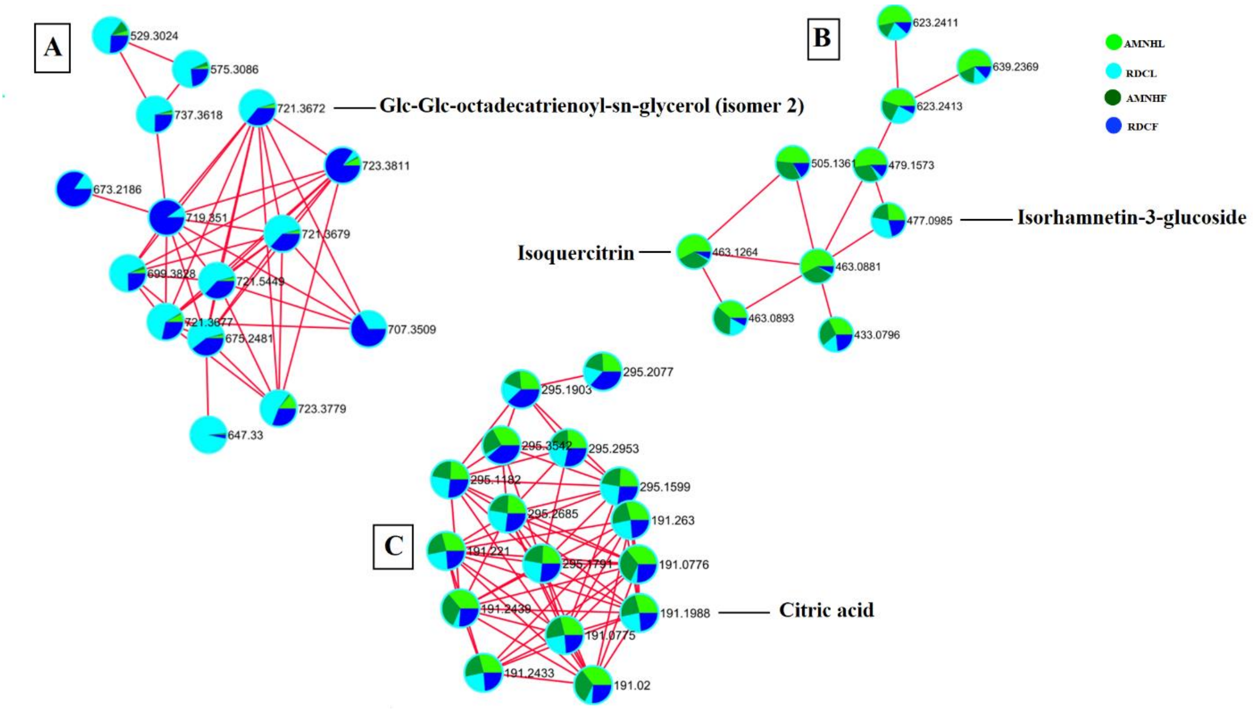

Figure S2: **Cannabis cultivar-specific and plant-tissue-specific metabolite differences**. A combined molecular network showing the relative quantification of some metabolites identified across the cultivars and their plant tissues. **Key**: Amnesia haze leaves (**AMNHL**), Amnesia haze flowers (**AMNHF**), Royal dutch cheese leaves (**RDCL**) and Royal dutch cheese flowers (**RDCF**). (A) Shows lipid-like molecules to be more abundant in the plant tissues of RDC, (B) shows flavonoids such as isoquercitrin to be more abundant in the tissues of AMNH while (C) shows a cluster of hydroxy acids such as citric acid to occur in similar amounts across both the cultivars.

Table S1: All putatively annotated metabolites (level 2 and 3 MSI) in the plant-tissues of Amnesia haze (AMNH) and Royal dutch cheese (RDC) Presence (✓) and absence (×) stating the distribution of metabolites and (*) denotes the abbreviations used in **Figure S2**.

No.	*m/z*	Rt	Adduct	Chemical formular	Fragment ions	Putative annotations	*Abbrev or alternative name	Superclass	Amnesia haze (AMNH)		Royal dutch cheese (RDC)	
									Leaves	Flowers	Leaves	Flowers
**1**	166.0854	2.422	$[M+H]^+$	$C_9H_{11}NO_2$	120,107,103	Phenylalanine	L-Phe	Organic acids and derivatives	✓	✓	✓	✓
**2**	205.0961	4.562	$[M+H]^+$	$C_{11}H_{12}N_2O_2$	170,159,143, 132,118	L-Tryptophan	L-Trp	Organic acids and derivatives	✓	✓	✓	✓
**3**	313.218	13.01	$[M-H]^-$	$C_{21}H_{30}O_2$	311,245,191, 136	Cannabidiol	CBD	lipid and lipid-like molecules	✓	✓	✓	✓
**4**	389.198	10.48	$[M-H]^-$	$C_{22}H_{32}O_6$	313,331,229, 205	Cannabitriolic acid	CBTA	lipid and lipid-like molecules	✓	✓	✓	✓
**5**	285.041	7.0955	$[M-H]^-$	$C_{22}H_{32}O_6$	175,151,133	Luteolin	Luteolin	Phenylpropanoids &polyketides	✓	✓	✓	✓
**6**	431.1	6.897	$[M-H]^-$, $[M+H]^+$	$C_{21}H_{20}O_{10}$	117,283,161	vitexin	Vit	Phenylpropanoids &polyketides	✓	✓	✓	✓
**7**	329.183	11.66	$[M-H]^-$	$C_{20}H_{26}O_4$	311,217,199, 107	Cannabidi-varinic acid	CBDVA	lipid and lipid-like molecules	✓	✓	✓	✓
**8**	357.208	16.50	$[M-H]^-$	$C_{22}H_{30}O_4$	311,245,191,1 36	Cannabidiolic acid	CBDA	lipid and lipid-like molecules	✓	✓	✓	✓
**9**	375.2188	11.412	$[M-H]^-$	$C_{22}H_{30}O_5$	357,273,179,1 35,122	6,7-epoxy-Cannabigerolic acid	6,7-Epoxy-CBGA	lipid and lipid-like molecules	✓	✓	✓	✓

**10**	315.0732	3.6545	$[M-H]^-$	$C_7H_6O_4$	152,109,108	2,5-Dihydroxybenz oic acid	2,5-Dihydro-acid	Organic acids and derivatives	✓	✕	✓	✕
**11**	721.3702	11.874	$[M-H]^-$	$C_{33}H_{56}O_{14}$	675,415,397, 277,235	Glc-Glc-octadecatrienoyl -sn-glycerol (isomer 2)	Glc-Glc-glycerol	lipid and lipid-like molecules	✓	✓	✓	✓
**12**	371.187	14.01	$[M-H]^-$	$C_{23}H_{32}O_4$	353,327,259, 191	Cannabidiolic acid monomethyl ether	CBDMA	lipid and lipid-like molecules	✓	✓	✓	✓
**13**	459.0943	8.783	$[M-H]^-$	$C_{22}H_{20}O_{11}$	113,283,268, 175	Oroxindin	Oroxindin	Phenylpropanoids &polyketides	✓	✕	✓	✕
**14**	312.13	7.6065	$[M-H]^-$	$C_{18}H_{19}NO_2$	148,176,190	Feruloyl-tyramine	Ferlulo-tyr	Phenylpropanoids &polyketides	✓	✓	✓	✓
**15**	447.09	6.547	$[M-H]^-$	$C_{21}H_{20}O_{11}$	445	Orientin	Orientin	Phenylpropanoids &polyketides	✓	✓	✓	✓
**16**	299.0566	7.8436	$[M-H]^-$	$C_{16}H_{12}O_6$	285,284	Diosmetin	Diosmetin	Phenylpropanoids &polyketides	✓	✕	✓	✕
**17**	577.1533	6.9476	$[M-H]^-$	$C_{27}H_{30}O_{14}$	413,293,173	Vitexin-2-O-rhamnoside	Vit-2-O-rhamn	Phenylpropanoids &polyketides	✓	✓	✓	✓
**18**	593.9265	6.329	$[M-H]^-$	$C_{27}H_{30}O_{15}$	503,473,383, 353	Vicenin 2	Vic-2	Phenylpropanoids &polyketides	✓	✕	✓	✕
**19**	452.2905	12.601	$[M+H]^+$	$C_{21}H_{44}NO_7P$	313	1-Palmitoyl-2-hydroxy-sn-glycero-3-phosphoethanol amine	1-Pal-hydr-gly-phos	lipid and lipid-like molecules	✓	✕	✓	✕
**20**	597.3066	12.99	$[M-H]^-$	$C_{27}H_{51}O_{12}P$	315,281,241,1 54	1-(9Z-Octadecenoyl)-sn-glycero-3-phospho-(1'-myo-inositol)	9Z-Octa-1-myo-inositol	lipid and lipid-like molecules	✓	✕	✓	✕

**21**	509.2904	13.126	$[M-H]^-$	$C_{24}H_{47}O_9P$	281,227,153	1-(9Z-Octadecenoyl)-sn-glycero-3-phospho-(1'-sn-glycerol)	9Z-Octa-1-sn-glycerol	lipid and lipid-like molecules	✓	✕	✓	✕
**22**	571.2912	12.864	$[M-H]^-$	$C_{25}H_{49}O_{12}P$	391,315,255, 241,153	1-Hexadecanoyl-sn-glycero-3-phospho-(1'-myo-inositol)	1-Hexa-phopho-1-myo-inositol	lipid and lipid-like molecules	✓	✕	✓	✕
**23**	493.1	6.611	$[M+FA-H]^-$	$C_{21}H_{20}O_{11}$	447,357,327	Luteolin-6-c-glucoside	Lut-6-glu	Phenylpropanoids &polyketides	✓	✓	✓	✓
**24**	483.2749	12.698	$[M-H]^-$	$C_{22}H_{45}O_9P$	255,245,227, 153	1-Hexadecanoyl-sn-glycero-3-phospho-(1'-sn-glycerol)	1-Hexa-phopho-1-sn-gly	lipid and lipid-like molecules	✓	✓	✓	✓
**25**	437.1823	9.908	$[M+H]^+$, $[M-H]^-$	$C_{26}H_{28}O_6$	313,289,299	Cannflavin A	Cannflavin A	Phenylpropanoids &polyketides	✓	✓	✓	✓
**26**	549.2557	7.307	$[M+FA-H]^-$	$C_{24}H_{40}O_{11}$	527	2-Cyclohexen-1-one, 3,5,5-trimethyl-4-[3-[(6-O-beta-D-xylopyranosyl-beta-D-glucopyranosyl) oxy]butyl]	2-cycl-1-one	lipid and lipid-like molecules	✓	✓	✓	✓
**27**	353.0889	5.113	$[M-H]^-$	$C_{16}H_{18}O_9$	179,191,135	Caffeoylquinic acid	Caffeo-qui-acid	Phenylpropanoids &polyketides	✓	✓	✓	✓
**28**	591.1811	7.4177	$[M-H]^-$	$C_{28}H_{32}O_{14}$	504,427,367, 326,307	2-O-rhamnosyl-swertisin	2-O-rhamn-swert	Phenylpropanoids &polyketides	✓	✕	✓	✕

**29**	327.2178	9.546	$[M\text{-}H]^-$	$C_{18}H_{32}O_5$	211,229,183, 171	9,12,13-trihydroxyoctadeca-10,15-dienoic acid	Trihydroxy-octa-dienic-acid	lipid and lipid-like molecules	✓	✓	✓	✓
**30**	367.1447	7.1555	$[M\text{-}H]^-$	$C_{17}H_{20}O_9$	191,173,193, 134	Feruloyl quinic acid (isomer of 887, 888)	Ferul-qui-acid	Phenylpropanoids &polyketides	✓	✓	✓	✓
**31**	433.0768	7.234	$[M\text{-}H]^-$	$C_{20}H_{18}O_{11}$	300,271,255,301	Guaijaverin	Guaijaverin	Phenylpropanoids &polyketides	✓	✓	✓	✓
**32**	609.146	7.01	$[M\text{-}H]^-$	$C_{27}H_{30}O_{16}$	300,271,151	Quercetin-3-rutinoside	Quercetin-3-rut	Phenylpropanoids &polyketides	✓	✓	✓	✓
**33**	709.3739	13.118	$[2M\text{-}2H+Na]^-$	$C_{18}H_{16}O_7$	343,299,301	Usnic acid	Usnic acid	Benzonoids	✕	✓	✕	✓
**34**	773.3542	11.315	$[2M\text{-}Na]^-$	$C_{19}H_{20}O_8$	375	Pseudo placodiolic acid	Pseudo-plac-acid	Benzonoids	✕	✓	✕	✓
**35**	577.1346	5.557	$[M\text{-}H]^-$	$C_{30}H_{26}O_{12}$	451,425,407,289,161	Procyanidin B1	Procyanidin B	Phenylpropanoids &polyketides	✓	✓	✓	✓
**36**	452.28	12.268	$[M\text{-}H]^-$	$C_{21}H_{44}NO_7P$	255,213	Phosphatidylethanolamine	Lyso 16:0	lipid and lipid-like molecules	✓	✓	✓	✓
**37**	477.1033	7.677	$[M\text{-}H]^-$	$C_{22}H_{22}O_{12}$	315,314,285 ,271	Isorhamnetin-3-glucoside	Isorhamn-3-glu	Phenylpropanoids &polyketides	✓	✓	✓	✓
**38**	289.07	5.976	$[M\text{-}H]^-$	$C_{15}H_{14}O_6$	287,245,137,125,109	(-)-Epicatechin	Epicatechin	Phenylpropanoids &polyketides	✓	✓	✓	✓
**39**	463.09	7.219	$[M\text{-}H]^-$	$C_{21}H_{20}O_{12}$	301,271,255	Isoquercitrin	Isoquercitrin	Phenylpropanoids &polyketides	✓	✓	✓	✓
**40**	579.1498	5.997	$2[M\text{-}H]^-$	$C_{15}H_{14}O_6$	289.,245,125	Catechin dimer	2XCatechin	Phenylpropanoids &polyketides	✓	✓	✓	✓

**41**	223.097	9.534	$[M-H]^-$	$C_{12}H_{16}O_4$	179,137	Olivetolic acid	Olivet-acid	Phenylpropanoids &polyketides	×	✓	×	✓
**42**	553.1658	9.634	$[M-H]^-$	$C_{25}H_{30}O_{14}$	391,373,347	Ligustrosidic acid	Ligus-acid	lipid and lipid-like molecules	×	✓	×	✓
**43**	403.1309	6.819	$[M-H]^-$	$C_{17}H_{24}O_{11}$	101,113,119,179,223	Oleoside 11-methyl ester	Oleo-methy-ester	lipid and lipid-like molecules	×	✓	×	✓
**44**	617.1451	6.685	$[M-Na]^+$	$C_{27}H_{30}O_{15}$	455,437,599	Saponarin	Saponarin	Phenylpropanoids &polyketides	✓	✓	✓	✓
**45**	601.1513	6.847	$[M-Na]^+$	$C_{30}H_{26}O_{12}$	583,481,455	Vitexin 2"-O-p-coumarate	Vit-2-O-coumarate	Phenylpropanoids &polyketides	✓	✓	✓	✓
**46**	524.3703	13.216	$[M+H]^+$	$C_{26}H_{54}NO_7P$	524,184,104	1-Octadecanoyl-sn-glycero-3-phosphocholine	1-Octadec-gly-phospho	lipid and lipid-like molecules	✓	✓	✓	✓
**47**	461.074	7.139	$[M-H]^-$	$C_{21}H_{18}O_{12}$	285,229	Kaempferol 3-glucuronoside	Kaemp-3-glu	Phenylpropanoids &polyketides	✓	✓	✓	✓
**48**	518.3212	12.699	$[M+Na]^+$	$C_{24}H_{50}NO_7P$	459,313,184,146,104	1-Hexadecanoyl-sn-glycero-3-phosphocholine	1-Hexadec-gly-phospho	lipid and lipid-like molecules	✓	✓	✓	✓
**49**	311.2076	13.98	$[M+H]^+$	$C_{21}H_{26}O_2$	223,121,109	Cannabinol	CBN	lipid and lipid-like molecules	✓	✓	✓	✓
**50**	341.2101	17.01	$[M+H]^+$	$C_{22}H_{29}O_3$	341,285,219,161	Tetrahydrocannabinolic acid-acylium ion ($\triangle^9$-THCA-1)	$\triangle^9$-THCA-1	lipid and lipid-like molecules	✓	✓	✓	✓
**51**	341.2104	12.04	$[M+H]^+$	$C_{22}H_{29}O_3$	229,219,161,117	Cannabidiolic acid- actrylium ion (CBDA-1)	CBDA-1	lipid and lipid-like molecules	✓	✓	✓	✓
**52**	359.2209	17.38	$[M+H]^+$	$C_{22}H_{30}O_4$	285,261,219,135	Canna-bicycliolic acid	CBLA	lipid and lipid-like molecules	✓	✓	✓	✓
**53**	463.3242	7.593	$[M+H]^+$	$C_{21}H_{18}O_{12}$	343,313,287	Breviscapine	Breviscapine	Phenylpropanoids &polyketides	✓	✓	✓	✓
**54**	338.412	14.336	$[M+H]^+$	$C_{22}H_{43}NO$	149,163,135,121,114, 107	Erucamide	Erucamide	lipid and lipid-like molecules	✓	×	✓	×

**55**	353.2079	12.151	$[M+H]^+$	$C_{21}H_{36}O_4$	261,243,173,149,121,107	Monolinolenin	Monolin	lipid and lipid-like molecules	✓	✓	✓	✓
**56**	315.231	15.43	$[M+H]^+$	$C_{21}H_{30}O_2$	193,135,123,109,107	$\triangle^9$−Tetrahydro cannabinol	$\triangle^9$-THC	lipid and lipid-like molecules	×	✓	×	✓
**57**	355.1897	15.93	$[M+H]^+$	$C_{22}H_{26}O_4$	337,313,253,219	Cannabinolic acid	CBDA	lipid and lipid-like molecules	✓	✓	✓	✓
**58**	522.3542	12.708	$[M+H]^+$	$C_{26}H_{52}NO_7P$	504,184,125,104	1-oleoyl-glycero-3-phosphochloline	1-Oleo-gly-3-phospho chloline	lipid and lipid-like molecules	×	✓	×	✓
**59**	601.13	5.271	$[M+Na]^+$	$C_{30}H_{26}O_{12}$	311,449,313	Procyanidin B2	Procy-B2	Phenylpropanoids &polyketides	×	✓	×	✓
**60**	796.45	14.938	$[M+H]^+$	$C_{42}H_{66}O_{14}$	735,517	Tenacissoside H	Tena-H	lipid and lipid-like molecules	✓	✓	✓	✓
**61**	395.26	11.614	$[M+H]^+$	$C_{19}H_{22}O_9$	377,359,352	Aloesin	Aloesin	Organoherocyclic compounds	×	✓	×	✓
**62**	413.2298	11.772	$[M+Na]^+$	$C_{23}H_{34}O_5$	395,377,231,149	Gitoxigenin	Gitox	lipid and lipid-like molecules	✓	✓	✓	✓
**63**	219.1733	9	$[M+Na]^+$	$C_{12}H_{20}O_2$	159,145,133,131,119,105	Linalyl butyrate	Lin-buty	Organic acids and derivatives	×	✓	×	✓
**64**	439.2295	7.331	$[M+H]^+$	$C_{21}H_{26}O_{10}$	277,259,203	Monnieriside G	Monn-G	Phenylpropanoids &polyketides	✓	✓	✓	✓
**65**	563.5502	13.1255	$2[M+H]^+$	$C_{18}H_{35}NO$	282,265,247,135,109	9-Octadecenamide	9-Octade	lipid and lipid-like molecules	×	✓	×	✓
**66**	182.0805	1.276	$[M+H]^+$	$C_9H_{11}NO_3$	147,136,123,119,107, 103	L-Tyrosine	L-Tyr	Organic acids and derivatives	✓	✓	✓	✓
**67**	369.110	11.712	$[M+H]^+$ $[M-H]^-$	$C_{21}H_{20}O_6$	313,298,165	Cannflavin B	Cannflavin-B	Phenylpropanoids &polyketides	✓	✓	✓	✓

**68**	563.1410	5.855	$[M\text{-}H]^-$	$C_{26}H_{28}O_{14}$	449,281,223, 163,135	Apigenin glucoside arabinoside	Api-glu-arab	Phenylpropanoids &polyketides	✓	✓	✓	✓
**69**	593.1520	6.553	$[M\text{-}H]^-$	$C_{27}H_{30}O_{15}$	473,429,327,3 09	Luteolin-7-rutinoside	Lut-7-rut	Phenylpropanoids &polyketides	✓	✓	✓	✓
**70**	609.1470	6.651	$[M\text{-}H]^-$	$C_{27}H_{30}O_{16}$	489,429,327, 309	Luteolin-glucoside-arabinoside	Lut-glu-arab	Phenylpropanoids &polyketides	✓	✓	✓	✓
**71**	299.057	9.09	$[M\text{-}H]^-$	$C_{16}H_{12}O_6$	285,284,256	Chrysoeriol	Chrysoeriol	Phenylpropanoids &polyketides	✕	✓	✕	✓
**72**	191.1980	1.02	$[M\text{-}H]^-$	$C_6H_8O_7$	147, 129, 87	Citric acid	Citric acid	Organic acids and derivatives	✓	✓	✓	✓

Table S2: Significant metabolic pathways in cannabis cultivars Amnesia haze (AMNH) and Royal dutch cheese (RDC) based on the KEGG IDs of metabolites identified in the leaves and flowers of the cultivars.

No.	Pathway	Total	Hits	*p*-value	Impact
**1**	Flavone and flavonol biosynthesis	10	2	8.4457E-4	0
**2**	Flavonoid biosynthesis	47	3	9.5604E-4	0.02046
**3**	Phenylalanine, tyrosine, and tryptophan biosynthesis	22	2	0.0042195	0.02002
**4**	Phenylpropanoid biosynthesis	46	2	0.017904	0.03161
**5**	Stilbenoid, diarylheptanoid and gingerol biosynthesis	8	1	0.03723	0.13235
**6**	Tyrosine metabolism	16	1	0.073265	0.10811
**7**	Tryptophan metabolism	28	1	0.12515	0.12307
**8**	Isoquinoline alkaloid biosynthesis	6	1	0.028036	0.5
**9**	Aminoacyl-tRNA biosynthesis	46	2	0.017904	0
**10**	Indole alkaloid biosynthesis	4	1	0.018766	0
**11**	Glycine, serine, and threonine metabolism	33	1	0.14602	0
**12**	Ubiquinone and other terpenoid-quinone biosynthesis	38	1	0.16646	0
**13**	Glucosinolate biosynthesis	65	1	0.26976	0

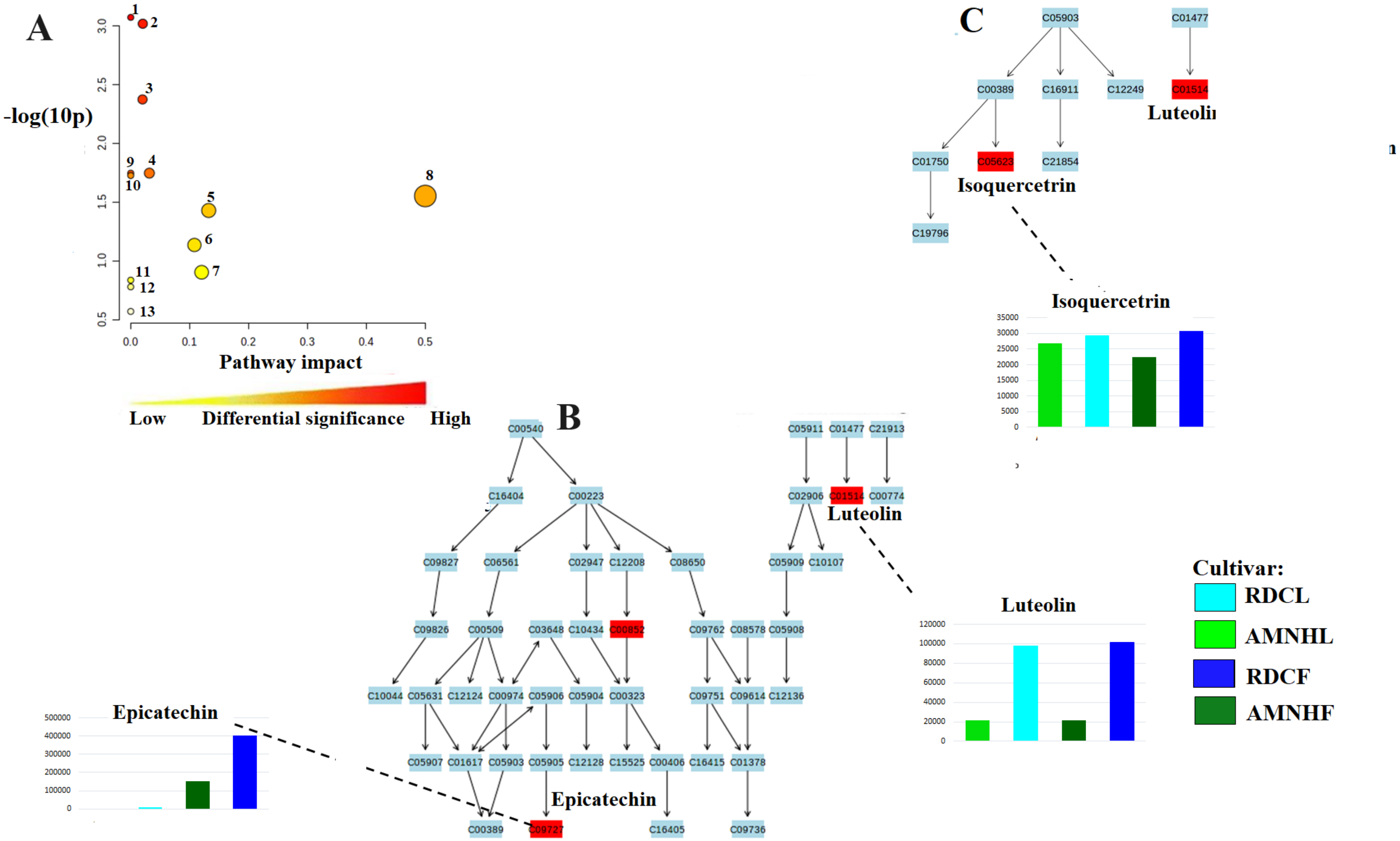

Figure S3: **Functional analysis**. Relative quantification of some metabolites identified across the leaves and flowers of AMNH and RDC cultivars. Summary of the metabolism pathways identified in the cannabis cultivars (A) where (B) represents the flavonoid and (C) flavone and flavanol biosynthesis pathways.

Table S3: Binding interactions identified for some cannabinoids and flavonoids ranked according to the docking score (binding affinity energy (kcal/mol), where a low docking score indicates good/significant interaction and a high score indicating bad/insignificant interaction between protein target and ligand).

Target and Ligand	Interacting Residues	Docking Score
**Cannabinoid receptor 2 (CB2) & $\triangle^9-$tetrahydrocannabinol ($\triangle^9$-THC)**	SER285 ,PHE281, VAL113, SER90	**-10.3 kcal/mol**
**Cannabinoid receptor 1 (CB1) & Cannabidiol (CBD)**	PHE108, PHE268, MET384, MET103, PHE170.	**-9.5 kcal/mol**
**Caspase 3 & Cannflavin A**	SER144, THR143, GLU196, TRY200, PRO206, TRY202, ARG167	**-9.3 kcal/mol**
**Cannabinoid receptor 1 (CB1) & $\triangle^9-$tetrahydrocannabinol ($\triangle^9$-THC)**	PHE108, PHE268, SER383, MET103, PHE170	**-9.2 kcal/mol**
**Cannabinoid receptor 2 (CB1) & Cannabidiol (CBD)**	HIS95, PHE91, PHE87, LYS109, PHE106, ILE110	**-9.2 kcal/mol**
**Mitogen-Activated Protein Kinase 1 (MEK1) & Apigenin**	TRP275, LEU294, LEU244, ILE243, ILE240, GLN236, PHE288, TRP212	**-8.3 kcal/mol**
**G-coupled receptor 55 (GPR55) & Cannabidiol**	VAL103, LEU96, LEU72, LEU100	**-7.8 kcal/mol**
**Fas receptor & Quercetin**	GLU270, ASN268, GLU163, LYS152, ASN154, SER153	**-6.6 kcal/mol**
**Vascular endothelial growth factor (VEGF) & Kaempferol**	LYS286, ASP276, ILE46, ILE48, SER50, ASP34, PHE36	**-6.6 kcal/mol**
**Tyrosine kinase & Luteolin**	ARG184, LYS182, TYR181, TRY209, GLY215, ILE193, SER194, ARG196	**-6.0 kcal/mol**

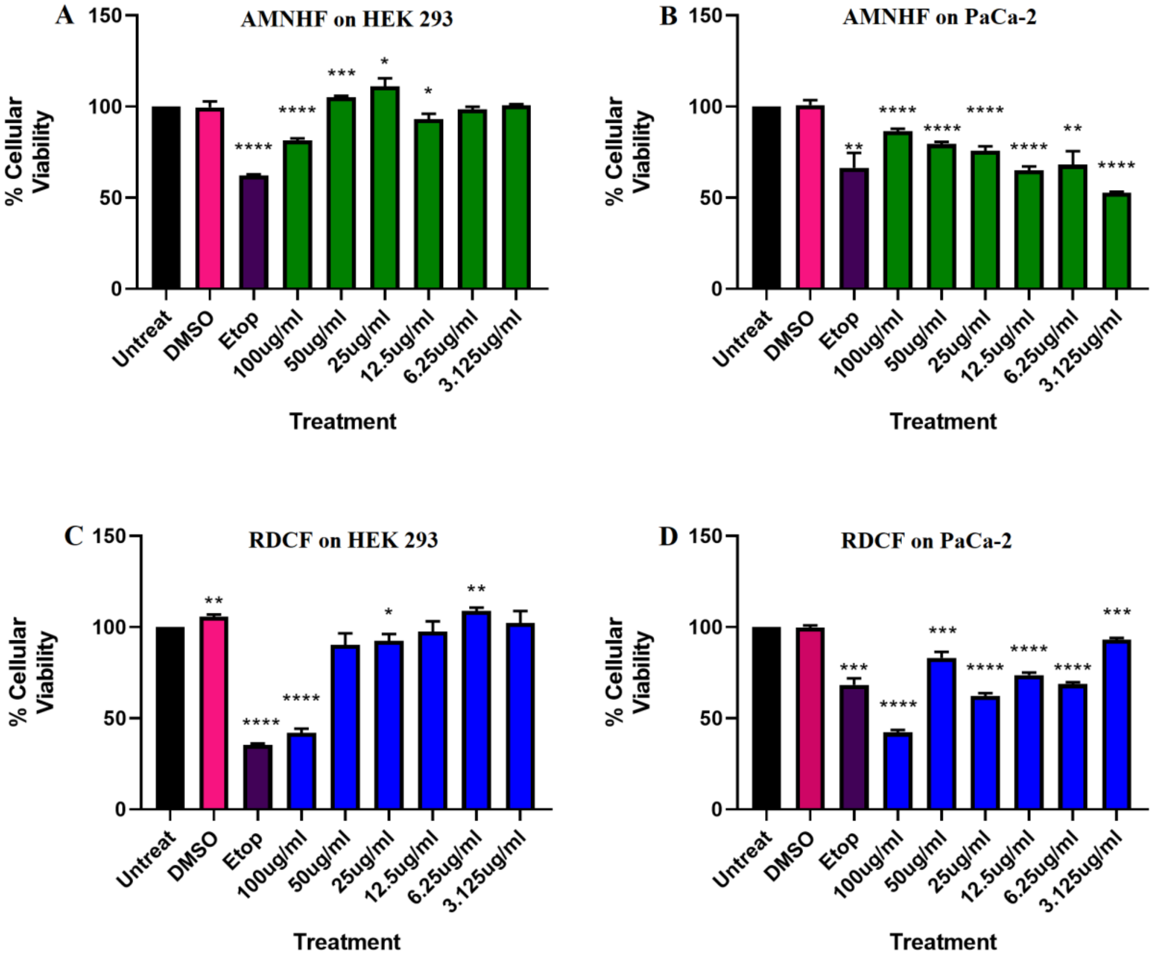

Figure S4: **Cell proliferation assessed by Alamar analysis**: (**A**) Percentage viability of HEK 293 (781.7 µg/ml) and (**B**) PaCa-2 cells (IC50 of 3 µg/ml) following the 24-hour treatment with AMNHF respectively. (C) Percentage viability of HEK 293 (IC50 92.48 µg/ml and (D) PaCa-2 cells (IC50 of 65.17 µg/ml) following the 24-hour treatment with RDCF respectively A T-test was performed to assess for statistical significance compared to the positive control (*P < 0.05, **P < 0.01 ***P < 0.001, ****P < 0.0001)).
